# Prognostic value of the lactate dehydrogenase-to-albumin ratio for predicting mortality in critically ill pediatric patients: a retrospective cohort study

**DOI:** 10.3389/fped.2025.1692874

**Published:** 2025-11-05

**Authors:** Ming Liu, Yunpeng Gou, Ping Yang

**Affiliations:** Department of Pediatric Surgery, Suining Central Hospital, Suining, Sichuan, China

**Keywords:** lactate dehydrogenase to albumin ratio (LAR), pediatric critical care, mortality prediction, prognostic biomarker, risk stratification

## Abstract

**Objective:**

Despite significant advances in pediatric intensive care, the early identification of high-risk critically ill children remains a persistent challenge. This study aimed to evaluate the association between the lactate dehydrogenase-to-albumin ratio (LAR) and mortality outcomes in critically ill pediatric patients.

**Methods:**

This retrospective cohort study analyzed data from the Pediatric Intensive Care (PIC) database (2010–2018) at the Children's Hospital of Zhejiang University School of Medicine. We included 8,782 critically ill patients aged ≥28 days with complete lactate dehydrogenase (LDH) and albumin (ALB) measurements. The LAR was calculated by dividing the serum LDH concentration by the ALB concentration. The primary outcome was 30-day in-hospital mortality, while the secondary outcome was in-hospital mortality. Multivariate Cox proportional hazards regression models were constructed with adjustments for demographic characteristics, clinical parameters, and laboratory variables.

**Results:**

After full adjustment for covariates, LAR remained significantly associated with mortality risk. Each 10 U/g increase in LAR was associated with a 3% higher risk of 30-day in-hospital mortality (HR = 1.03, 95% CI: 1.01–1.04, *P* = 0.005) and a 4% higher risk of in-hospital mortality (HR = 1.04, 95% CI: 1.03–1.06, *P* < 0.001). Compared to the lowest tertile, the highest tertile had a significantly higher mortality risk (30-day in-hospital mortality: HR = 3.72, 95% CI: 2.50–5.54; in-hospital mortality: HR = 2.68, 95% CI: 1.86–3.87; both *P* < 0.001). Receiver operating characteristic (ROC) analysis revealed that LAR's discriminative performance (AUC = 0.771 for 30-day in-hospital mortality; AUC = 0.763 for in-hospital mortality) outperformed that of either LDH or ALB alone.

**Conclusion:**

Elevated LAR independently predicts an increased mortality risk in critically ill pediatric patients. As an easily calculated ratio derived from routine laboratory parameters, LAR represents a valuable prognostic tool for risk stratification in the pediatric intensive care setting.

## 1 Introduction

The management of critically ill children constitutes a major challenge in global public health, particularly in pediatric intensive care units, where mortality rates significantly exceed those in general pediatric wards (1). Despite considerable advances in monitoring technologies and therapeutic interventions in recent years, mortality among these vulnerable patients remains a pressing concern, especially in high-risk subpopulations (2, 3). Therefore, early identification of children with elevated mortality risk continues to represent a crucial yet challenging objective in pediatric critical care.

Various biomarkers, including lactate dehydrogenase (LDH) and albumin (ALB), are widely utilized to evaluate disease severity and prognosis in critically ill children (4–7). However, single biomarkers often inadequately reflect the complex pathophysiology of critical illness in children, limiting their accuracy in predicting mortality (8). This underscores the need for more robust prognostic indicators. The lactate dehydrogenase-to-albumin ratio (LAR), a novel composite marker that quantifies both cellular damage and nutritional/inflammatory status simultaneously, has been shown to correlate with adverse outcomes in various critical conditions (9–11). Nevertheless, evidence supporting the utility of the LAR in predicting mortality among pediatric intensive care patients remains limited.

Consequently, this study sought to examine the association between LAR and mortality outcomes, including both 30-day and overall in-hospital mortality, in a large cohort of critically ill pediatric patients using data from the Pediatric Intensive Care (PIC) database. Our findings aim to improve the early risk stratification of high-risk patients and potentially inform clinical decision-making strategies in pediatric critical care settings.

## 2 Materials and methods

### 2.1 Data source

Data for this study were obtained from the PIC database, an open-access, single-center resource developed by the Children's Hospital of Zhejiang University School of Medicine, China (http://pic.nbscn.org). This retrospective database contains anonymized electronic health records from 12,881 pediatric patients across 13,941 hospitalizations who received care in five intensive care units: the general intensive care unit (GICU), the cardiac intensive care unit (CICU), the neonatal intensive care unit (NICU), the surgical intensive care unit (SICU), and the pediatric intensive care unit (PICU). The database encompasses data spanning from 2010 to 2018. The hospital serves as the largest comprehensive pediatric medical center in Zhejiang Province, with over 1,900 beds, 119 of which are dedicated to intensive care. The PIC database contains comprehensive clinical information, including laboratory measurements, diagnostic data, physical assessments, medication administrations, surgical procedures, and admission/discharge records. Data usage was approved by the Institutional Ethics Review Committee of Zhejiang University School of Medicine's Children's Hospital, with individual informed consent waived due to the retrospective study design and complete anonymization of all patient records.

### 2.2 Study population

**Figure 1:**
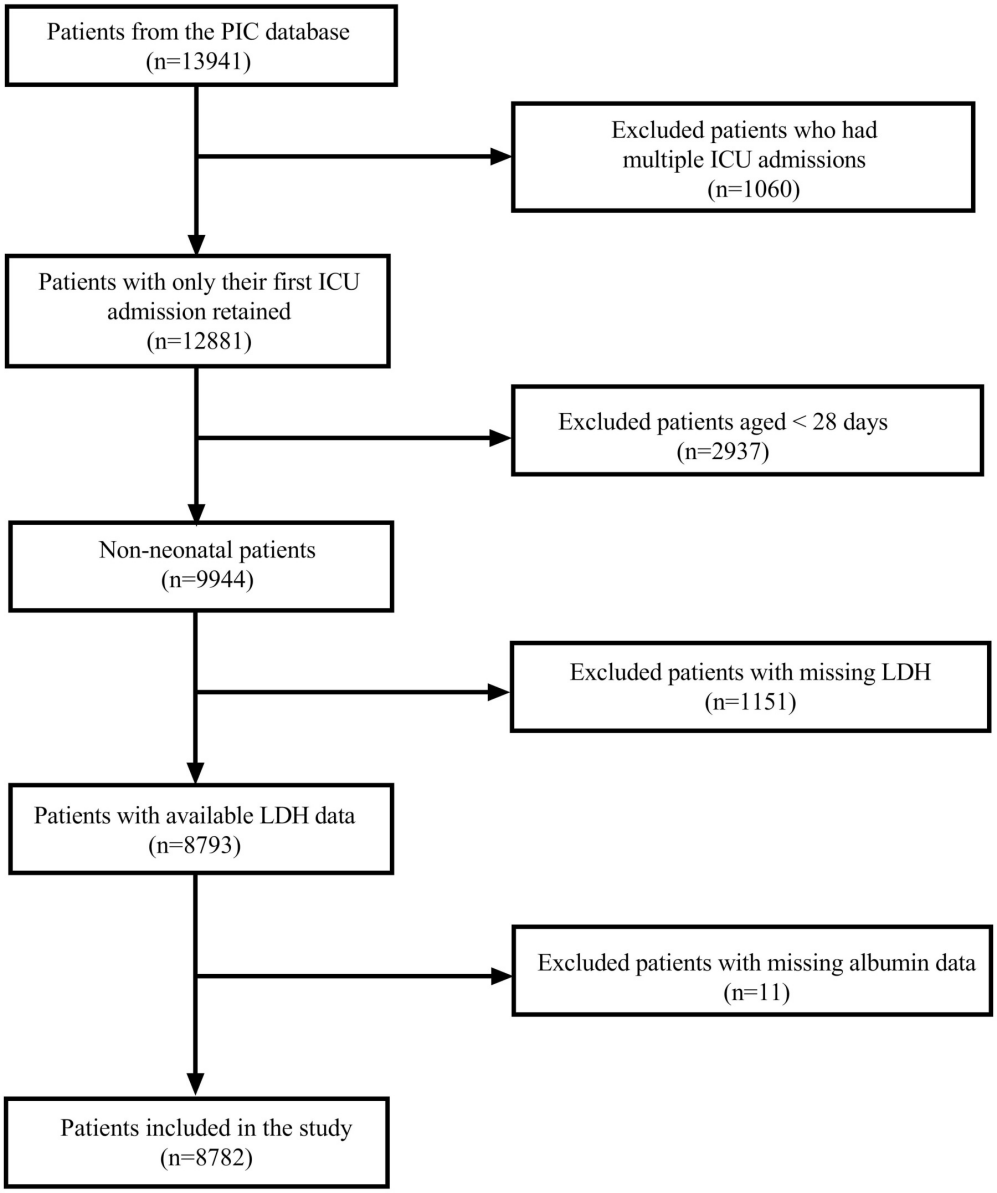
Flowchart of patient selection from the PIC database.

The initial dataset comprised 13,941 hospitalizations from the PIC database. For patients with multiple ICU admissions, only the first admission was retained; this excluded 1,060 subsequent hospitalizations, yielding 12,881 unique patients. We then excluded 2,937 neonatal patients (aged <28 days), leaving 9,944 non-neonatal patients for analysis. Among these patients, 1,151 were further excluded due to missing LDH data. This resulted in 8,793 patients with available LDH measurements. After the final exclusion of 11 patients with missing ALB data, our analytical cohort comprised 8,782 patients (Figure 1).

### 2.3 Clinical data extraction

Clinical data were extracted from the PIC database following institutional approval. Extracted variables included demographic characteristics (age, gender, and ethnicity), ICU type, vital signs at admission, primary diagnosis upon ICU admission, surgical procedure types, length of hospital stay, and in-hospital mortality. Diagnoses were coded using the International Classification of Diseases, Tenth Revision (ICD-10) system. We documented the use of vasoactive medications, including dobutamine, dopamine, adrenaline, phenylephrine, isoprenaline, and norepinephrine. Laboratory parameters were obtained from the first blood sample collected after ICU admission and included white blood cell count (WBC), hemoglobin (HGB), platelet count (PLT), C-reactive protein (CRP), triglycerides (TG), total cholesterol (TC), total bilirubin (TBIL), alanine aminotransferase (ALT), LDH, ALB, serum creatinine (Scr), glucose (Glu), sodium (Na), calcium (Ca), lactate, and pH.

### 2.4 Exposure variable and outcome measures

In this study, the LAR served as the exposure variable and was calculated by dividing the serum LDH level by the ALB level. The primary outcome was 30-day in-hospital mortality, defined as death from any cause occurring during the hospitalization and within 30 days of admission. Patients were followed from admission until death, discharge alive, or 30 days post-admission, whichever occurred first. Patients discharged alive before day 30 were censored at the time of discharge in survival analyses; post-discharge vital status was not ascertained in this study. The secondary outcome was in-hospital mortality, defined as death occurring during the hospitalization period regardless of cause.

### 2.5 Statistical analysis

Study participants were divided into three groups based on LAR tertiles. Due to the non-normal distribution of continuous variables, the data were presented as the median (interquartile range) and were compared across groups using the Kruskal–Wallis rank sum test. Categorical variables were presented as frequencies (percentages) and compared using the chi-square test.

To examine the association between LAR and mortality outcomes, multivariable Cox proportional hazards regression models were performed, with results presented as hazard ratios (HRs) and 95% confidence intervals (CIs). LAR was analyzed both as a continuous variable (per 10 U/g increment) and as a categorical variable using tertiles (T1, T2, and T3). Potential confounders were selected based on clinical relevance and existing literature (12–16). Three progressive models were constructed: Model 1 (unadjusted); Model 2 (adjusted for age, gender, and ethnicity); and Model 3 (fully adjusted for age, gender, ethnicity, primary diagnosis, temperature, use of vasopressors, and laboratory parameters including WBC, HGB, PLT, TG, TC, TBIL, ALT, Scr, Glu, Na, Ca, and lactate).

To evaluate the robustness of the association between LAR and mortality outcomes, stratified analyses were conducted across various subgroups, including age categories, gender, primary diagnosis, and vasopressor use, using the fully adjusted model (Model 3). All covariates were adjusted, except for the stratification variable itself. The consistency of these associations and potential effect modification was examined by formal interaction testing, with statistical significance determined by the *P*-value for the interaction term. It is important to note that the subgroup analyses should be interpreted as exploratory. The statistical power to detect a significant association varies across subgroups due to differences in sample size and the number of outcome events. Therefore, results from subgroups with a limited number of events should be interpreted with caution.

Fully adjusted smoothed curve fitting was employed to explore the dose-response relationship between LAR and both 30-day and overall in-hospital mortality outcomes. Survival probabilities across LAR tertiles (T1, T2, and T3) were compared using Kaplan–Meier curves, and statistical significance was assessed by a log-rank test. Receiver operating characteristic (ROC) curves were generated to evaluate and compare the prognostic value of LAR, LDH, and ALB for mortality, and the area under the curve (AUC) was calculated to quantify the predictive performance.

All statistical analyses were conducted using Empower Stats (http://www.empowerstats.com, X&Y Solutions, Inc., CA, USA) and R software (version 4.4.0; https://www.R-project.org). Statistical significance was defined as a two-sided *p* value less than 0.05.

## 3 Results

### 3.1 Baseline characteristics of the study population

A total of 8,782 participants were included and stratified into three groups according to LAR tertiles (T1–T3), as shown in Tables 1, 2. Significant differences were observed in the majority of baseline characteristics across the tertiles. Patients in the highest LAR group (T3) were younger, had higher heart rates and body temperatures, and exhibited lower systolic and diastolic blood pressures compared to those in the lower tertiles (all *P* < 0.001). The distribution of ethnicity and ICU types also varied significantly, with a greater proportion of patients being of Han ethnicity in the T1 and T2 groups, and higher rates of PICU and NICU admissions observed in the T3 group. Regarding laboratory parameters, T3 patients had higher levels of white blood cells, CRP, ALT, TBIL, LDH, and lactate, along with lower levels of HGB, PLT, ALB, TC, and Ca (all *P* < 0.001). The primary diagnoses and types of surgical procedures also differed significantly among the tertiles. T3 patients had more respiratory, hematological, trauma, and sepsis cases, along with more gastrointestinal and general surgeries. Median hospital length of stay increased progressively with rising LAR. Notably, both the 30-day and the overall in-hospital mortality rates increased markedly across the LAR tertiles, with the highest rates observed in the T3 group (*P* < 0.001 for both outcomes). No significant differences were found in gender distribution, TG levels, Na levels, Glu levels, or vasopressor use among the groups.

**Table 1:** Clinical characteristics and outcomes of study participants stratified by LAR tertiles.

Characteristic/outcome	Total	LAR tertile			*P*-value
		T1 (LAR < 6.39)	T2 (6.39 ≤ LAR < 9.19)	T3 (LAR ≥ 9.19)	
Participants	8,782	2,926	2,928	2,928	
Age (years)	1.49 (0.42–4.80)	3.71 (1.04–7.97)	1.01 (0.33–2.75)	1.02 (0.29–3.33)	<0.001
Gender, *n* (%)					0.606
Female	3,861 (43.96%)	1,308 (44.70%)	1,280 (43.72%)	1,273 (43.48%)	
Male	4,921 (56.04%)	1,618 (55.30%)	1,648 (56.28%)	1,655 (56.52%)	
Ethnicity, *n* (%)					<0.001
Han ethnicity	8,697 (99.03%)	2,906 (99.32%)	2,911 (99.42%)	2,880 (98.36%)	
Miao ethnicity	20 (0.23%)	1 (0.03%)	2 (0.07%)	17 (0.58%)	
Yi ethnicity	7 (0.08%)	2 (0.07%)	1 (0.03%)	4 (0.14%)	
Hui ethnicity	6 (0.07%)	1 (0.03%)	1 (0.03%)	4 (0.14%)	
Tujia ethnicity	11 (0.13%)	5 (0.17%)	4 (0.14%)	2 (0.07%)	
Buyei ethnicity	14 (0.16%)	5 (0.17%)	2 (0.07%)	7 (0.24%)	
Other ethnicity	27 (0.31%)	6 (0.21%)	7 (0.24%)	14 (0.48%)	
ICU types, *n* (%)					<0.001
GICU	1,828 (20.82%)	602 (20.57%)	494 (16.87%)	732 (25.00%)	
PICU	1,805 (20.55%)	384 (13.12%)	495 (16.91%)	926 (31.63%)	
NICU	421 (4.79%)	27 (0.92%)	146 (4.99%)	248 (8.47%)	
SICU	2,401 (27.34%)	952 (32.54%)	880 (30.05%)	569 (19.43%)	
CICU	2,327 (26.50%)	961 (32.84%)	913 (31.18%)	453 (15.47%)	
Temperature (℃)	36.90 (36.50–37.00)	36.80 (36.50–37.00)	36.80 (36.50–37.00)	37.00 (36.60–37.20)	<0.001
Heart rate (bpm)	126.00 (110.00–138.00)	116.00 (100.00–130.00)	128.00 (114.00–140.00)	132.00 (118.00–148.00)	<0.001
SpO2 (%)	99.00 (92.30–99.70)	99.30 (95.73–99.80)	99.10 (94.40–99.70)	98.40 (86.60–99.60)	<0.001
SBP (mmHg)	99.00 (89.00–-109.00)	102.00 (94.00–112.00)	98.00 (88.00–107.00)	98.00 (86.00–108.00)	<0.001
DBP (mmHg)	57.00 (49.00–66.00)	60.00 (52.00–69.00)	56.00 (47.00–64.00)	56.00 (46.00–65.00)	<0.001
Primary diagnosis, *n* (%)					<0.001
Cardiovascular	1,929 (21.97%)	709 (24.23%)	774 (26.43%)	446 (15.23%)	
Congenital	1,053 (11.99%)	414 (14.15%)	376 (12.84%)	263 (8.98%)	
Digestive	946 (10.77%)	331 (11.31%)	330 (11.27%)	285 (9.73%)	
Hematological	367 (4.18%)	65 (2.22%)	74 (2.53%)	228 (7.79%)	
Neoplasm	525 (5.98%)	225 (7.69%)	158 (5.40%)	142 (4.85%)	
Neurological	1,184 (13.48%)	459 (15.69%)	383 (13.08%)	342 (11.68%)	
Respiratory	1,100 (12.53%)	204 (6.97%)	366 (12.50%)	530 (18.10%)	
Trauma	462 (5.26%)	82 (2.80%)	127 (4.34%)	253 (8.64%)	
Urinary	432 (4.92%)	237 (8.10%)	133 (4.54%)	62 (2.12%)	
Sepsis	164 (1.87%)	26 (0.89%)	30 (1.02%)	108 (3.69%)	
Others	620 (7.06%)	174 (5.95%)	177 (6.05%)	269 (9.19%)	
Surgical procedures, *n* (%)					<0.001
Cardiac	2,005 (39.24%)	772 (36.87%)	808 (42.19%)	425 (38.60%)	
Gastrointestinal	579 (11.33%)	179 (8.55%)	229 (11.96%)	171 (15.53%)	
General	96 (1.88%)	23 (1.10%)	33 (1.72%)	40 (3.63%)	
Hepatobiliary	251 (4.91%)	87 (4.15%)	96 (5.01%)	68 (6.18%)	
Neurosurgical	971 (19.00%)	414 (19.77%)	355 (18.54%)	202 (18.35%)	
Oncological	428 (8.38%)	194 (9.26%)	138 (7.21%)	96 (8.72%)	
Otorhinolaryngology	155 (3.03%)	75 (3.58%)	66 (3.45%)	14 (1.27%)	
Respiratory	24 (0.47%)	4 (0.19%)	12 (0.63%)	8 (0.73%)	
Thoracic	329 (6.44%)	210 (10.03%)	93 (4.86%)	26 (2.36%)	
Urological	149 (2.92%)	88 (4.20%)	46 (2.40%)	15 (1.36%)	
Others	123 (2.41%)	48 (2.29%)	39 (2.04%)	36 (3.27%)	
Use of vasopressors, *n* (%)					0.286
No	5,693 (64.83%)	1,869 (63.88%)	1,896 (64.75%)	1,928 (65.85%)	
Yes	3,089 (35.17%)	1,057 (36.12%)	1,032 (35.25%)	1,000 (34.15%)	
LOS hospital (days)	12.00 (7.00–19.00)	11.00 (7.00–16.75)	12.00 (8.00–19.00)	12.50 (6.00–23.00)	<0.001
30-day in-hospital mortality, *n* (%)					<0.001
No	8,392 (95.56%)	2,891 (98.80%)	2,861 (97.71%)	2,640 (90.16%)	
Yes	390 (4.44%)	35 (1.20%)	67 (2.29%)	288 (9.84%)	
In-hospital mortality, *n* (%)					<0.001
No	8,335 (94.91%)	2,883 (98.53%)	2,848 (97.27%)	2,604 (88.93%)	
Yes	447 (5.09%)	43 (1.47%)	80 (2.73%)	324 (11.07%)	

Continuous variables are presented as the median (interquartile range) and compared using the Kruskal–Wallis rank sum test due to their non-normal distribution. Categorical variables are presented as numbers (percentages) and compared using the chi-square test.

LAR, lactate dehydrogenase-to-albumin ratio; GICU, general intensive care unit; PICU, pediatric intensive care unit; NICU, neonatal intensive care unit; SICU, surgical intensive care unit; CICU, cardiac intensive care unit; SpO2, pulse oxygen; bpm, beats per minute; SBP, systolic blood pressure; DBP, diastolic blood pressure; LOS, length of stay.

**Table 2:** Laboratory parameters of study participants stratified by LAR tertile.

Characteristic/outcome	Total	LAR tertile			*P*-value
		T1 (LAR < 6.39)	T2 (6.39 ≤ LAR < 9.19)	T3 (LAR ≥ 9.19)	
WBC (×10^9^/L)	9.01 (6.86–12.06)	8.17 (6.44–10.35)	9.39 (7.27–12.14)	9.88 (6.95–14.30)	<0.001
HGB (g/L)	116.00 (102.00–126.00)	122.00 (112.00–130.00)	115.00 (103.00–125.00)	107.00 (91.00–121.00)	<0.001
PLT (×10^9^/L)	318.00 (237.00–401.00)	316.00 (255.00–386.00)	344.00 (273.00–426.00)	279.00 (155.00–394.75)	<0.001
CRP (mg/L)	3.00 (0.71–7.00)	0.71 (0.35–4.00)	2.00 (0.71–5.00)	5.00 (0.71–21.00)	<0.001
TG (mmol/L)	1.11 (0.75–1.65)	1.08 (0.75–1.57)	1.12 (0.77–1.63)	1.12 (0.73–1.74)	0.056
TC (mmol/L)	3.62 (2.94–4.32)	3.84 (3.32–4.43)	3.68 (3.07–4.32)	3.21 (2.41–4.11)	<0.001
TBIL (μmol/L)	7.50 (5.10–12.60)	7.20 (5.30–10.20)	7.10 (5.10–12.00)	8.60 (4.90–19.60)	<0.001
ALT (U/L)	20.00 (13.00–34.00)	15.00 (11.00–23.00)	20.00 (14.00–31.00)	29.00 (17.00–70.50)	<0.001
LDH (U/L)	306.00 (253.00–418.00)	238.00 (209.00–262.00)	308.00 (282.00–340.00)	515.50 (414.00–782.00)	<0.001
ALB (g/L)	41.90 (37.20–45.20)	44.30 (41.73–46.70)	42.10 (38.70–45.10)	37.20 (32.00–41.60)	<0.001
Scr (μmol/L)	43.00 (37.00–52.00)	46.00 (39.10–55.00)	41.00 (36.00–47.00)	43.00 (35.00–53.00)	<0.001
Glu (mmol/L)	5.50 (4.70–6.60)	5.50 (4.80–6.40)	5.50 (4.70–6.52)	5.50 (4.50–6.90)	0.799
Na (mmol/L)	136.00 (134.00–139.00)	136.00 (134.00–139.00)	136.00 (134.00–139.00)	136.00 (133.00–139.00)	0.18
Ca (mmol/L)	2.37 (2.23–2.49)	2.41 (2.32–2.51)	2.42 (2.30–2.52)	2.23 (2.07–2.38)	<0.001
Lactate (mmol/L)	1.60 (1.10–2.60)	1.50 (1.00–2.10)	1.60 (1.00–2.50)	1.90 (1.20–3.30)	<0.001
PH	7.39 (7.32–7.43)	7.40 (7.35–7.44)	7.39 (7.34–7.44)	7.37 (7.30–7.43)	<0.001

All continuous variables are presented as the median (interquartile range) and compared using the Kruskal–Wallis rank sum test due to their non-normal distribution.

LAR, lactate dehydrogenase-to-albumin ratio; WBC, white blood cell count; HGB, hemoglobin; PLT, platelet count; CRP, c-reactive protein; TG, triglycerides; TC, total cholesterol; TBIL, total bilirubin; ALT, alanine aminotransferase; LDH, lactate dehydrogenase; ALB, serum albumin; Scr, serum creatinine; Glu, glucose; Na, sodium; Ca, calcium.

### 3.2 Association of LAR with 30-day and overall in-hospital mortality

Table 3 presents the association between LAR and both 30-day and overall in-hospital mortality. After full adjustment for potential confounders (Model 3), each 10 U/g increase in LAR was associated with a 3% increased risk of 30-day in-hospital mortality (HR = 1.03, 95% CI: 1.01–1.04, *P* = 0.005). Compared with the lowest tertile (T1), patients in the highest tertile (T3) had a 272% increased risk of 30-day in-hospital mortality (HR = 3.72, 95% CI: 2.50–5.54, *P* < 0.001). A significant upward trend in mortality risk was evident across increasing LAR tertiles (*P* for trend < 0.001). Similar associations were found for in-hospital mortality: each 10 U/g increase in LAR was associated with a 4% increased risk (HR = 1.04, 95% CI: 1.03–1.06, *P* < 0.001), and the highest tertile showed a 168% increased risk of in-hospital mortality compared to the lowest tertile (HR = 2.68, 95% CI: 1.86–3.87, *P* < 0.001), with a significant upward trend (*P* for trend < 0.001).

**Table 3:** Association of LAR with 30-day and overall in-hospital mortality.

Event type	Incidence of events	Model 1 HR (95% CI), *P* value	Model 2 HR (95% CI), *P* value	Model 3 HR (95% CI), *P* value
30-day in-hospital mortality				
LAR (per 10 U/g)	390/8,782	1.05 (1.04, 1.05), <0.001	1.05 (1.04, 1.06), <0.001	1.03 (1.01, 1.04), 0.005
LAR tertiles				
T1	35/2,926	Reference	Reference	Reference
T2	67/2,928	1.93 (1.28, 2.90), 0.002	2.12 (1.40, 3.21), <0.001	1.65 (1.07, 2.56), 0.025
T3	288/2,928	8.60 (6.05, 12.21), <0.001	9.30 (6.50, 13.31), <0.001	3.72 (2.50, 5.54), <0.001
*P* for trend		<0.001	<0.001	<0.001
In-hospital mortality				
LAR (per 10 U/g)	447/8,782	1.05 (1.04, 1.06), <0.001	1.05 (1.04, 1.06), <0.001	1.04 (1.03, 1.06), <0.001
LAR tertiles				
T1	43/2,926	Reference	Reference	Reference
T2	80/2,928	1.72 (1.19, 2.50), 0.004	1.94 (1.33, 2.83), <0.001	1.50 (1.01, 2.25), 0.046
T3	324/2,928	6.16 (4.47, 8.49), <0.001	6.81 (4.91, 9.45), <0.001	2.68 (1.86, 3.87), <0.001
*P* for trend		<0.001	<0.001	<0.001

Model 1: unadjusted model.

Model 2: adjusted for: age, gender, and ethnicity.

Model 3: adjusted for: age, gender, ethnicity, primary diagnosis, temperature, use of vasopressors, WBC, HGB, PLT, TG, TC, TBIL, ALT, Scr, Glu, Na, Ca, and lactate.

LAR, lactate dehydrogenase-to-albumin ratio; HR, hazard ratio; CI, confidence interval; WBC, white blood cell count; HGB, hemoglobin; PLT, platelet count; TG, triglycerides; TC, total cholesterol; TBIL, total bilirubin; ALT, alanine aminotransferase; Scr, serum creatinine; Glu, glucose; Na, sodium; Ca, calcium.

**Figure 2:**
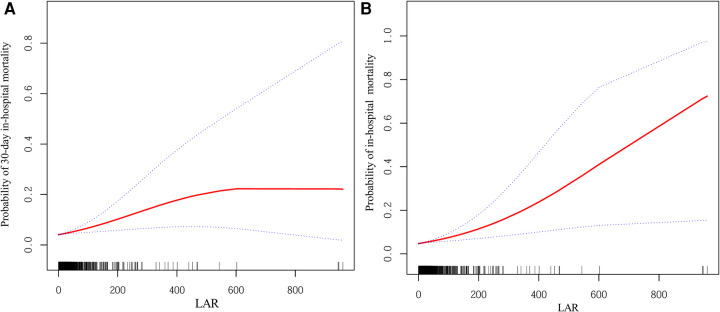
Smoothed curves showing the association between LAR and the probability of mortality. **(A)** Probability of 30-day in-hospital mortality. **(B)** Probability of in-hospital mortality. The red line represents the fitted curve, and the blue dashed lines indicate the 95% confidence intervals. Adjusted for: age, gender, ethnicity, primary diagnosis, temperature, use of vasopressors, WBC, HGB, PLT, TG, TC, TBIL, ALT, Scr, Glu, Na, Ca, and lactate. LAR, lactate dehydrogenase-to-albumin ratio; WBC, white blood cell count; HGB, hemoglobin; PLT, platelet count; TG, triglycerides; TC, total cholesterol; TBIL, total bilirubin; ALT, alanine aminotransferase; Scr, serum creatinine; Glu, glucose; Na, sodium; Ca, calcium.

Figures 2A,B present the smoothed curve-fitting analyses of the association between LAR and the probability of 30-day and overall in-hospital mortality, respectively, after full adjustment for confounding factors (Model 3). Both figures demonstrate an increase in mortality probability with higher LAR values. In Figure 2A, the probability of 30-day in-hospital mortality reaches a plateau at higher LAR levels, whereas in Figure 2B, the probability continues to increase across the observed range.

**Figure 3:**
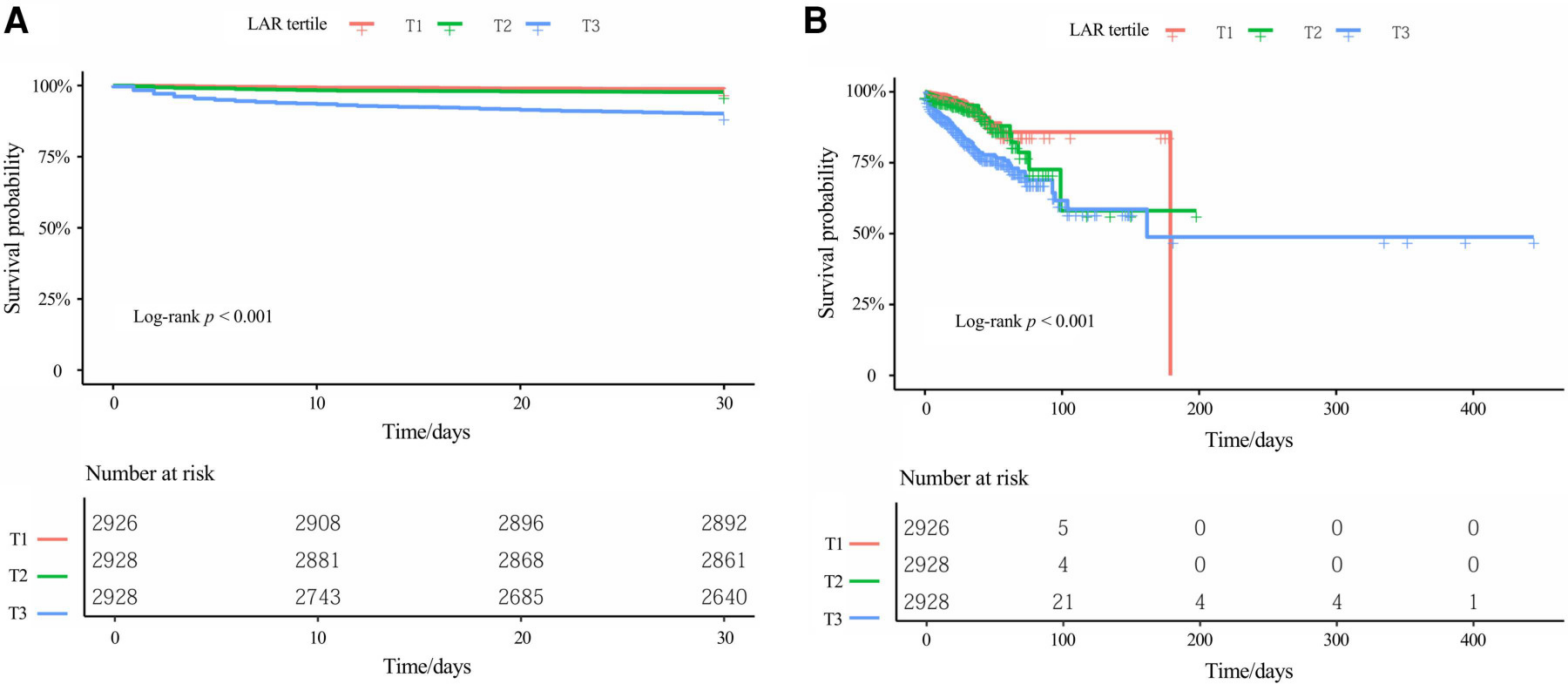
Kaplan–meier survival curves for **(A)** 30-day in-hospital mortality and **(B)** in-hospital mortality, stratified by LAR tertiles. Kaplan–Meier curves display the probability of survival over time for different patient groups, with each downward step representing an event (death). The log-rank test was used to compare survival distributions across groups, with *P* < 0.001 indicating statistically significant differences among the three tertiles. The table below each Kaplan-Meier curve displays the number of patients still being followed (at risk) at each specified time point. LAR, lactate dehydrogenase-to-albumin ratio.

To assess survival probabilities across LAR tertiles over time, Kaplan–Meier survival analyses were conducted (Figure 3). For 30-day survival (Figure 3A), patients in the highest LAR tertile (T3) exhibited the lowest survival probabilities throughout the follow-up period, with statistically significant differences observed among the three groups (log-rank *P* < 0.001). A similar pattern was observed for in-hospital survival (Figure 3B), where the T3 group consistently demonstrated poorer survival compared to the T1 and T2 groups, and the separation between the survival curves widened over time (log-rank *P* < 0.001).

### 3.3 Subgroup analysis and interaction analysis

Subgroup and interaction analyses based on the fully adjusted model (Model 3) were conducted to evaluate the consistency of the association between LAR and both 30-day and overall in-hospital mortality (Table 4). Overall, the positive association between higher LAR and increased mortality risk remained robust across the majority of subgroups. However, adolescents aged ≥12 years showed a significant inverse association (HR = 0.50, *P* < 0.001 for 30-day in-hospital mortality; HR = 0.58, *P* < 0.001 for in-hospital mortality). Despite this variation, formal tests indicated no statistically significant interactions for age, gender, or primary diagnosis (all *P* for interaction > 0.05). Notably, vasopressor use emerged as a significant effect modifier for both 30-day in-hospital mortality (*P* for interaction = 0.013) and in-hospital mortality (*P* for interaction < 0.001), with stronger associations observed among patients receiving vasopressors. For in-hospital mortality, the interaction with primary diagnosis reached borderline significance (*P* for interaction = 0.050). It should be noted that results for the urinary subgroup could not be reliably interpreted due to inestimable HRs and extremely wide CIs.

**Table 4:** Subgroup analyses and interaction effects for the association of LAR (per 10 U/g) with 30-day and overall in-hospital mortality.

Stratification factor	HR (95% CI)	*P*-value	*P* for interaction
30-day in-hospital mortality			
Age (years)			0.716
<1	1.04 (1.00, 1.07)	0.053	
1–3	1.03 (0.99, 1.06)	0.105	
3–6	1.04 (0.99, 1.10)	0.127	
6–12	1.12 (1.03, 1.22)	0.011	
≥12	0.50 (0.42, 0.60)	<0.001	
Gender			0.243
Female	1.02 (0.99, 1.06)	0.181	
Male	1.03 (1.01, 1.05)	0.016	
Primary diagnosis			0.193
Cardiovascular	1.19 (0.82, 1.73)	0.367	
Congenital	0.88 (0.68, 1.12)	0.296	
Digestive	1.01 (0.94, 1.07)	0.879	
Hematological	1.05 (0.98, 1.12)	0.180	
Neoplasm	1.12 (1.00, 1.26)	0.051	
Neurological	1.08 (0.95, 1.22)	0.225	
Respiratory	1.01 (0.92, 1.12)	0.780	
Trauma	0.79 (0.53, 1.17)	0.242	
Urinary	0.14 (0.00, Inf)	1.000	
Sepsis	1.02 (1.00, 1.05)	0.083	
Others	1.11 (1.02, 1.20)	0.014	
Use of vasopressors			0.013
No	1.02 (0.98, 1.06)	0.381	
Yes	1.03 (1.01, 1.05)	0.003	
In-hospital mortality			
Age (years)			0.263
<1	1.08 (1.04, 1.12)	<0.001	
1–3	1.04 (1.02, 1.07)	0.001	
3–6	1.07 (1.01, 1.13)	0.014	
6–12	1.12 (1.04, 1.22)	0.003	
≥12	0.58 (0.49, 0.70)	<0.001	
Gender			0.086
Female	1.05 (1.02, 1.08)	0.001	
Male	1.04 (1.02, 1.06)	<0.001	
Primary diagnosis			0.050
Cardiovascular	1.21 (0.86, 1.71)	0.268	
Congenital	0.98 (0.76, 1.27)	0.873	
Digestive	1.04 (0.99, 1.10)	0.101	
Hematological	1.03 (0.97, 1.10)	0.313	
Neoplasm	1.15 (0.99, 1.33)	0.077	
Neurologic	1.14 (0.98, 1.32)	0.087	
Respiratory	1.04 (0.94, 1.14)	0.444	
Trauma	1.10 (0.81, 1.51)	0.538	
Urinary	11.33 (0.00, Inf)	1.000	
Sepsis	1.02 (1.00, 1.05)	0.060	
Others	1.09 (1.00, 1.19)	0.041	
Use of vasopressors			<0.001
No	1.04 (0.99, 1.08)	0.119	
Yes	1.04 (1.02, 1.06)	<0.001	

Age, gender, ethnicity, primary diagnosis, temperature, use of vasopressors, WBC, HGB, PLT, TG, TC, TBIL, ALT, Scr, Glu, Na, Ca, and lactate were all adjusted except the stratification variable.

LAR, lactate dehydrogenase-to-albumin ratio; HR, hazard ratio; CI, confidence interval; WBC, white blood cell count; HGB, hemoglobin; PLT, platelet count; TG, triglycerides; TC, total cholesterol; TBIL, total bilirubin; ALT, alanine aminotransferase; Scr, serum creatinine; Glu, glucose; Na, sodium; Ca, calcium.

### 3.4 ROC analysis

**Figure 4:**
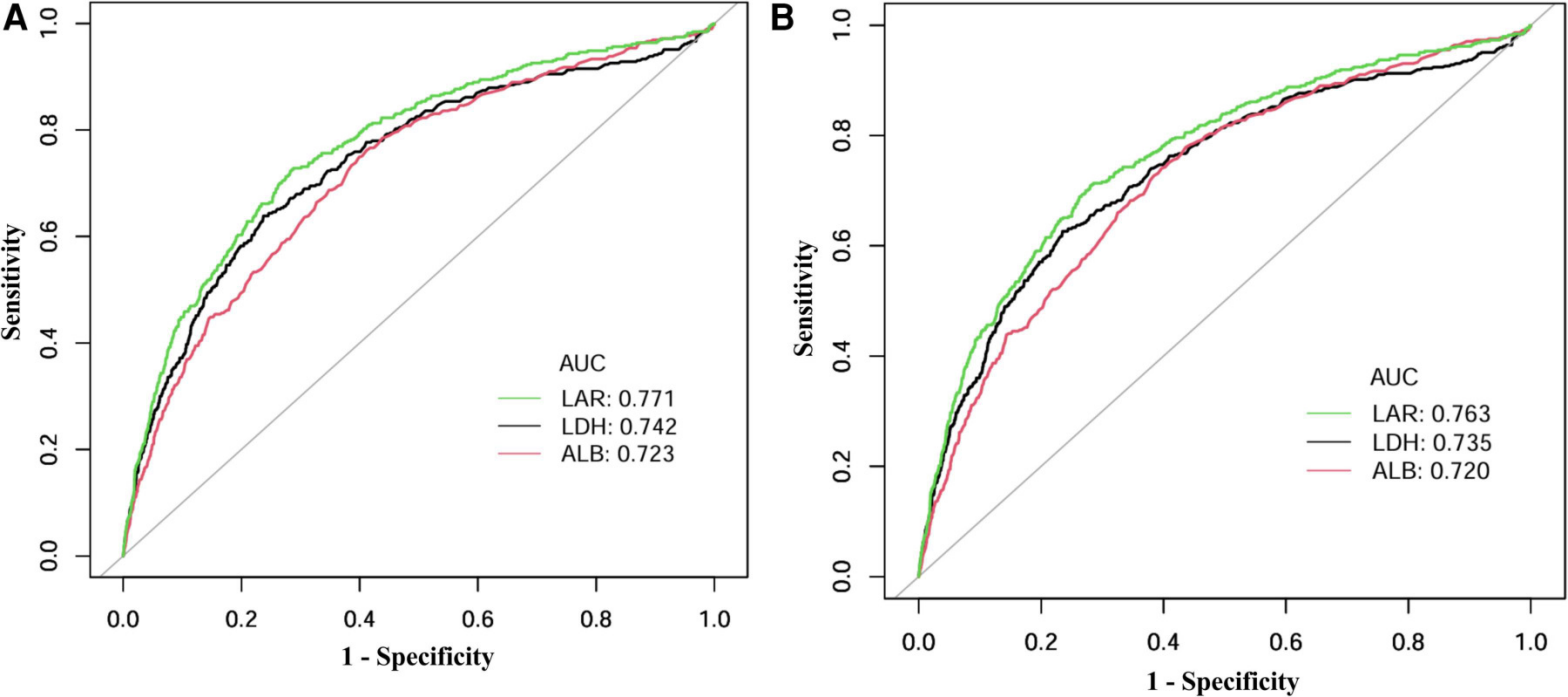
ROC curves for predicting **(A)** 30-day in-hospital mortality and **(B)** in-hospital mortality by LAR, LDH, and ALB. ROC curves plot sensitivity (true positive rate) against 1-specificity (false positive rate) to illustrate the predictive performance of biomarkers across all possible thresholds. The area under the curve (AUC) provides an aggregate measure of predictive accuracy, ranging from 0.5 (no discrimination) to 1.0 (perfect discrimination), with higher values indicating better discriminative ability. ROC, receiver operating characteristic; LAR, lactate dehydrogenase-to-albumin ratio; LDH, lactate dehydrogenase; ALB, serum albumin.

ROC analysis showed that LAR had superior discriminative performance for predicting mortality compared to LDH or ALB alone (Figure 4). For 30-day in-hospital mortality (Figure 4A), LAR achieved the highest AUC of 0.771 (95% CI: 0.745–0.796), with an optimal cutoff of 9.76, yielding a sensitivity of 72.3% and a specificity of 71.8%. In comparison, LDH (AUC = 0.742, 95% CI: 0.714–0.769) and ALB (AUC = 0.723, 95% CI: 0.697–0.750) showed lower discriminative ability. Similarly, for in-hospital mortality prediction (Figure 4B), LAR achieved the highest performance with an AUC of 0.763 (95% CI: 0.739–0.788) and the same optimal cutoff of 9.76, providing a sensitivity of 70.9% and specificity of 72.0%. Both LDH (AUC = 0.735, 95% CI: 0.709–0.762) and ALB (AUC = 0.720, 95% CI: 0.696–0.745) showed lower predictive power.

## 4 Discussion

In this large-scale retrospective analysis of 8,782 critically ill children from the PIC database, we examined the association between LAR and mortality outcomes. Our findings reveal that elevated LAR values are significantly associated with an increased risk of both 30-day and overall in-hospital mortality. After full adjustment for potential confounders, we found that each 10 U/g increase in LAR was associated with a 3% increased risk of 30-day in-hospital mortality. It was also associated with a 4% increased risk of in-hospital mortality. Patients in the highest LAR tertile showed a substantially higher mortality risk compared to those in the lowest tertile, with HRs of 3.72 for 30-day in-hospital mortality and 2.68 for in-hospital mortality. Importantly, LAR demonstrated superior discriminative ability compared to either LDH or ALB alone, suggesting its potential value as a more comprehensive prognostic marker in pediatric critical care settings.

Our findings are consistent with those of several previous studies examining LAR's prognostic value in critical care settings. For example, Xia et al. (17) found that LAR was significantly associated with in-hospital mortality in acute heart failure patients using the MIMIC-III database, reporting a 9% increase in mortality risk per unit increase in LAR. Similarly, Ye et al. (18) identified LAR as an independent predictor of 30-day in-hospital mortality in cardiac arrest patients, with an optimal threshold of 15.50, notably higher than our cutoff of 9.76, likely reflecting physiological differences between pediatric and adult populations. Both studies employed similar methodological approaches, using retrospective cohort designs with large databases and multivariate regression models that confirmed LAR's independent predictive value.

The prognostic value of LAR extends beyond cardiovascular conditions. Zhang et al. (11) validated LAR as a significant mortality predictor in ARDS patients, while Deng et al. (19) confirmed its utility in acute kidney injury, identifying a nonlinear relationship between LAR and in-hospital mortality, which is similar to our findings. Notably, Zhong et al. (20) and Chu et al. (10) demonstrated LAR's predictive capacity for 28-day mortality in ischemic stroke patients. The optimal cutoffs were substantially lower (approximately 0.55), highlighting the disease-specific nature of LAR thresholds. In oncological settings, He et al. (21) and Xu et al. (22) established LAR's association with long-term survival in breast and bladder cancer, respectively, indicating its applicability beyond acute critical care contexts.

The superior performance of LAR compared to its individual components can be attributed to several underlying pathophysiological mechanisms. LDH is an intracellular enzyme that is released during tissue damage and cellular injury. It functions as a marker of hypoxia, inflammation, and oxidative stress (23, 24)—conditions that are prevalent in critically ill children. Su et al. (25), examining a large adult ICU cohort, and Dong et al. (26), studying patients with COVID-19, demonstrated that elevated LDH levels independently correlate with increased mortality across diverse conditions. Meanwhile, hypoalbuminemia reflects malnutrition, increased capillary permeability, inflammation, and impaired protein synthesis (27, 28). Zhang et al. (29) identified a U-shaped association between ALB and PICU mortality, while Chen et al. (30) established that low ALB independently predicts heart failure mortality. By combining these parameters, LAR simultaneously captures both tissue damage and systemic inflammatory/nutritional status, thereby providing a more comprehensive assessment of critical illness severity.

The optimal LAR cutoff value of 9.76, as determined by our ROC analysis, has significant important clinical utility for risk stratification in pediatric critical care. The consistency of this cutoff value in predicting both 30-day in-hospital and overall in-hospital mortality underscores its robustness. This threshold, with balanced sensitivity (72.3%) and specificity (71.8%) for predicting 30-day in-hospital mortality, can serve as an early warning trigger to identify high-risk patients upon PICU admission, prompting clinicians to initiate more intensive monitoring and consider early escalation of supportive therapies. Compared to comprehensive severity scores such as PELOD, PRISM, or PIM, which require the collection of multiple physiological and laboratory parameters and involve complex calculations, LAR offers the practical advantages of simplicity and rapid availability, thanks to two routine biochemical tests that are typically available in the early phases of critical care. Rather than replacing existing mortality prediction systems, LAR could function as a complementary adjunctive tool in initial risk assessment. This would be particularly valuable in resource-limited settings or during early triage, when rapid decision-making is crucial. However, this threshold, which was derived from a single-center retrospective cohort, requires external validation in diverse pediatric populations before it can be implemented in routine clinical practice.

A notable finding from our subgroup analysis was the significant interaction effect observed with vasopressor use for both 30-day in-hospital mortality (*P* for interaction = 0.013) and, more prominently, in-hospital mortality (*P* for interaction < 0.001). In patients receiving vasopressors, LAR (per 10 U/g) showed a stronger association with mortality, with significant HRs (30-day in-hospital mortality: HR = 1.03, *P* = 0.003; in-hospital mortality: HR = 1.04, *P* < 0.001). Conversely, this association was attenuated and non-significant in patients not requiring vasopressor support. This interaction suggests that LAR may serve as a particularly valuable prognostic marker in hemodynamically compromised children requiring cardiovascular support. We hypothesize that the requirement of vasopressor use identifies states of circulatory failure and tissue hypoperfusion, where subsequent ischemia/reperfusion injury may accelerate cellular lysis and elevate LDH levels (31). Additionally, critical illness-associated inflammation and capillary leak syndrome, which are typically more pronounced in shock states requiring vasopressors, may substantially reduce ALB levels (32–34). These factors may synergistically enhance both LAR values and their association with mortality outcomes. However, our study only identified this statistical interaction without establishing causality or confirming underlying mechanisms. Further experimental and clinical studies are needed to elucidate the precise physiological processes underlying this association and to validate LAR's enhanced prognostic value specifically in vasopressor-dependent pediatric patients.

An intriguing finding emerged from the subgroup analysis of children aged 12 years or older [*n* = 455, with 17/455 (3.74%) 30-day deaths and 18/455 (3.96%) in-hospital deaths], where a higher LAR was unexpectedly associated with lower mortality risk (HR = 0.50, 95% CI: 0.42–0.60 for 30-day in-hospital mortality; HR = 0.58, 95% CI: 0.49–0.70 for in-hospital mortality), contrary to the positive association observed in younger age groups. While statistically significant, this inverse relationship should be interpreted with caution for several reasons. First, the stability of the estimates may be affected by the low event rates in the ≥12-year subgroup (3.74% for 30-day in-hospital mortality; 3.96% for in-hospital mortality), raising the possibility of chance findings or of the model being sensitive to influential observations. Second, adolescents may exhibit distinct clinical profiles compared with younger children, which could introduce unmeasured confounding factors beyond the adjustments applied in our model. Third, physiological and developmental factors during puberty—including changes in lactate metabolism, hepatic albumin synthesis, and overall organ reserve—may modify how LDH and ALB reflect illness severity in this age range. Fourth, residual and unmeasured confounding cannot be excluded in this subgroup, as our multivariable model was optimized for the entire cohort and may not fully account for adolescent-specific risk factors. Notably, the interaction test for in-hospital mortality was not significant (*P* for interaction = 0.263), suggesting that the apparent inverse association may not reflect a qualitatively different age effect at the population level. Taken together, these findings should be considered exploratory and require confirmation in independent adolescent cohorts before any clinical implications can be inferred.

Several limitations of this study deserve acknowledgment. First, our exclusion criteria eliminated neonatal patients (aged less than 28 days), patients with multiple ICU admissions (we retained only the first admission), and those with missing LDH or ALB data, potentially restricting the generalizability of our findings to these specific populations. By excluding subsequent ICU admissions, we may have overlooked important patterns in patients requiring repeated critical care. Second, as a single-center investigation conducted at the Children's Hospital of Zhejiang University School of Medicine, where over 99% of patients were of Han ethnicity, our results may be limited in generalizability to broader pediatric populations due to differences in ethnic, geographic, and healthcare systems. The clinical course of children may also be influenced by genetic, environmental, and socioeconomic factors that were not fully captured in this study. Therefore, validation in diverse, multicenter, and international cohorts is necessary. Third, the retrospective observational design precludes establishing causality between LAR and mortality, demonstrating only association. Fourth, despite adjusting for multiple measurable confounders, including demographics, clinical parameters, and laboratory indicators, unmeasured factors such as detailed nutritional status, socioeconomic variables, and established pediatric severity scores (e.g., PRISM, PIM, and PELOD), along with genetic predisposition and variations in critical care management practices, could have influenced the outcomes. Consequently, residual confounding cannot be fully excluded. Fifth, our analysis relied exclusively on single measurements of LDH and albumin obtained from admission blood samples. These biomarkers can change substantially during the PICU stay in response to disease progression and therapeutic interventions; however, our study only captured initial admission values. This approach may have limited the predictive power of LAR by failing to capture dynamic changes that distinguish patients with improving vs. deteriorating clinical trajectories. Future prospective studies should evaluate whether serial LAR measurements or dynamic trajectories offer superior predictive performance compared to single admission values. Sixth, the subgroup analyses, while comprehensive, should be interpreted with caution due to considerations of statistical power. This is exemplified by an unexpected inverse association between LAR and mortality in the ≥12-year subgroup, which may be sensitive to the limited number of outcome events in this and other smaller strata. More broadly, the reduced sample size and event count within various subgroups limit the ability to detect significant associations and the precision of the estimates. Therefore, these subgroup findings, including the inverse association in adolescents, should be viewed as exploratory and require validation in larger, dedicated cohorts. Seventh, ascertainment of the primary outcome was limited to in-hospital events, as post-discharge vital status was not available. Therefore, patients discharged alive within 30 days were censored, potentially leading to an underestimation of the true 30-day mortality. Nevertheless, the robust association between LAR and overall in-hospital mortality underscores the consistency of our main finding. Future validation in cohorts with post-discharge follow-up is recommended. Despite these limitations, this study provides robust evidence through its large sample size and comprehensive analytical approach supporting LAR's prognostic utility in pediatric critical care.

In conclusion, this study demonstrates that an elevated LAR level independently predicts both 30-day and overall in-hospital mortality in critically ill pediatric patients, with greater prognostic accuracy compared to either marker alone. As a simple ratio derived from routinely available laboratory tests, LAR could serve as a valuable prognostic indicator for risk stratification in pediatric intensive care settings. Future multicenter studies are warranted to validate these findings in diverse populations and to assess the utility of serial LAR measurements for monitoring disease progression and treatment response.

## Data Availability

The raw data supporting the conclusions of this article will be made available by the authors, without undue reservation.
